# Diagnostic performance of tomosynthesis plus synthetic mammography versus full‐field digital mammography with or without tomosynthesis in breast cancer screening: A systematic review and meta‐analysis

**DOI:** 10.1002/ijc.35217

**Published:** 2024-10-12

**Authors:** Wasim Hamad, Michael J. Michell, Jonathan P. Myles, Fiona J. Gilbert, Yan Chen, Huajie Jin, John Loveland, Mark Halling‐Brown, Keshthra Satchithananda, Juliet Morel, Rema Wasan, Caroline Taylor, Nisha Sharma, Alexandra Valencia, Will Teh, Faisal Majid, Ronald M De Visser, Asif Iqbal, Stephen W. Duffy

**Affiliations:** ^1^ Wolfson Institute of Population Health Queen Mary University of London London UK; ^2^ Breast Radiology Department and National Breast Training Centre Kings College Hospital London UK; ^3^ Department of Radiology University of Cambridge School of Clinical Medicine Cambridge UK; ^4^ Applied Vision Research Centre University of Nottingham Nottingham UK; ^5^ Scientific Computing Royal Surrey County Hospital Guildford UK; ^6^ The Jarvis Breast Centre Guildford UK; ^7^ Breast Screening Unit Leeds Teaching Hospital NHS Trust Leeds UK; ^8^ Avon Breast Screening Unit Bristol Breast Care Centre Bristol UK; ^9^ North London Breast Screening Service Edgware Community Hospital London UK; ^10^ Sandwell and Walsall Breast Screening Service Sandwell & West Birmingham NHS Trust Birmingham UK

**Keywords:** breast cancer, digital breast tomosynthesis, screening, synthetic mammogram

## Abstract

Digital breast tomosynthesis (DBT) with full‐field digital mammography (FFDM) exposes women to a higher radiation dose. A synthetic 2D mammogram (S2D) is a two‐dimensional image constructed from DBT. We aim to evaluate the S2D performance when used alone or combined with DBT compared to FFDM alone or with DBT. Studies were included if they recruited screening participants and reported on S2D performance. Studies were excluded if they included symptomatic patients, imaging was for diagnostic purposes, or if participants had a breast cancer history. Meta‐analyses for cancer detection rates (CDR) and Specificities were conducted where available. Differences in the performance of imaging modalities were calculated within individual studies, and these were pooled by meta‐analysis. Out of 3241 records identified, 17 studies were included in the review and 13 in the meta‐analysis. The estimated combined difference in CDRs per thousand among individual studies that reported on DBT plus S2D vs. FFDM and those reporting on DBT plus S2D versus DBT plus FFDM was 2.03 (95% CI 0.81–3.25) and − 0.15 (95% CI −1.17 to 0.86), respectively. The estimated difference in percent specificities was 1.13 (95% CI −0.06 to 2.31) in studies comparing DBT plus S2D and FFDM. In studies comparing DBT plus S2D and DBT plus FFDM, the estimated difference in specificities was 1.08 (95% CI 0.59–1.56). DBT plus S2D showed comparable accuracy to FFDM plus DPT and improved cancer detection to FFDM alone. Integrating S2D with DBT in breast cancer screening is safe and preserves performance.

## What's new?

Digital breast tomosynthesis (DBT), when combined with full‐field digital mammogram (FFDM), exhibits relatively high accuracy in breast cancer diagnosis. The combination, however, increases radiation exposure. This review evaluated the performance of DBT plus synthesized 2D (S2D), derived from reconstructed tomosynthesis images, as an alternative to DBT + FFDM. DBT + S2D was found to have a higher cancer detection rate compared to FFDM alone and exhibited superior specificity relative to DBT + FFDM. CDR, however, was similar for DBT + S2D and DBT + FFDM. DBT and S2D perform similarly to DBT combined with FFDM, while mitigating increased radiation exposure, suggesting that it is a safe and effective alternative.

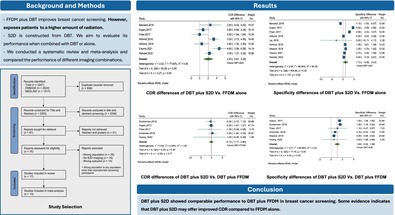

## 1 INTRODUCTION

Full‐field digital mammogram (FFDM) has been used for breast cancer screening for many years. However, recently, the use of digital breast tomosynthesis (DBT) plus FFDM has been found to improve breast cancer detection, sensitivity, and specificity mainly due to improved performance in the presence of overlapping breast tissue.^1^, ^2^, ^3^, ^4^ Published evidence concluded that DBT is superior to FFDM in breast cancer screening; hence, DBT was recommended in several guidelines as the standard tool for breast cancer screening.^5^, ^6^ In many cases, DBT is combined with FFDM to improve diagnostic performance, potentially exposing screened women to more than double the radiation dose.^4^, ^7^, ^8^ The development of a synthetic 2D mammogram (S2D), derived from the reconstructed tomosynthesis images, provides the reader with a 2D overview that can be used with the DBT images without the need for the additional exposure needed for FFDM. In their latest publications, the European Commission Initiative on Breast Cancer makes a conditional recommendation for the use of DBT plus S2D and the American College of Radiology does not make a strong recommendation either in favor or against S2D.^9^, ^10^ However, increasing evidence supports the integration of S2D with DBT to replace FFDM and avoid increasing radiation as much.^8^, ^11^, ^12^ Changing to DBT, with or without S2D raises issues of radiological practice. For example, at incidence screens, there is well‐developed expertise in comparison of current FFDM images with previous FFDM images. There is also the issue of time taken to read the images and come to a decision. For these reasons and others, there is a lack of agreement on the impact on accuracy of primary screening tests as replacing FFDM with S2D.^13^ The objective of this study is to compare the diagnostic performance of S2D plus DBT to FFDM alone or combined with DBT. We aimed to estimate and compare the cancer detection rate (CDR) and specificity across the different imaging combinations.

## 2 METHODS

### 2.1 Review strategy

A systematic review and meta‐analysis were conducted to evaluate the diagnostic performance of S2D plus DBT and compare it to the performance of FFDM alone or in combination with DBT for the detection of breast cancer in breast cancer screening settings. The search strategy was developed after reviewing the literature on S2D, FFDM, and DBT in breast cancer screening. To maximize the sensitivity of the search, we assessed the search methodologies of other reviews and used both MeSH and keyword search strategies.

A systematic electronic search was conducted on MEDLINE and EMBASE to identify primary studies published in or prior to January 2023. The search terms used are presented in Supplementary Appendix 1. References and citations of included papers were assessed for eligibility. No limitations on language, publication date, or study size were applied. There were no limitations on the study design.

### 2.2 Eligibility of studies

Included studies had to evaluate the performance of S2D either alone or in combination with other modalities for the detection of breast cancer in asymptomatic participants recruited from breast cancer screening settings. Studies had to include at least one of the diagnostic performance measures‐ CDR, false positive rate (FPR), sensitivity, or specificity. The reference standard for cancer diagnosis was histopathology. Studies were excluded if they did not perform S2D alone or combined with other modalities if symptomatic participants were included, if imaging was conducted due to diagnostic purposes, or if the study did not report on any of the eligible diagnostic performance measures. PRISMA flow chart was used to record the number of articles found and the subsequent selection process of eligible studies. Studies identified from different databases were merged in Endnote 20, and then duplicates were identified and removed. Title and abstract screening were conducted using Rayyan.^14^ Title and abstract screenings were conducted by WH. In the event of uncertainty, an expert reviewer was consulted (SWD). Two reviewers (WH and SWD) conducted full‐text screening of records and the screening was blinded, with an overall agreement of 83% and a kappa statistic of 0.60. Inclusion decisions and reasons for exclusion were reported using an Excel sheet. Disagreements between reviewers were resolved through consensus.

### 2.3 Information extracted

Data on publication year, study objectives, study design, population size, inclusion and exclusion criteria for each study, health care settings, reference standards, age, symptomatology, and imaging modalities were extracted. Extracted diagnostic measures included CDR, FPR, sensitivity, specificity, and 95% confidence intervals (CIs) on these.

### 2.4 Statistical analysis

Meta‐analysis was conducted using random effects models fitted with restricted maximum likelihood using the meta suite in the statistical computer package STATA.^15^, ^16^ Our primary meta‐analyses were of the within‐study comparisons of detection modalities. In addition, we derived meta‐analysis estimates of performance measures (see Supplementary Appendix 2). It should be noted that performance measures from the latter analysis are not directly comparable between modalities as each overall estimate is based on different populations.

If a study did not report standard errors (SE) or CIs but reported sufficient data in the publication to calculate them, these were calculated by the reviewers. This is demonstrated by example in Supplementary Appendix 3.

Specificities were also calculated from the published data in studies that reported only FPR. Meta‐analysis was conducted for CDRs and specificities for DBT plus S2D, FFDM alone, and DBT plus FFDM.

Measures of the performance of S2D alone and sensitivities were not pooled due to the low number of studies that reported on them. Studies where CIs and SE were not available or without enough data available to calculate them were excluded from the meta‐analysis.

## 3 RESULTS

**FIGURE 1:**
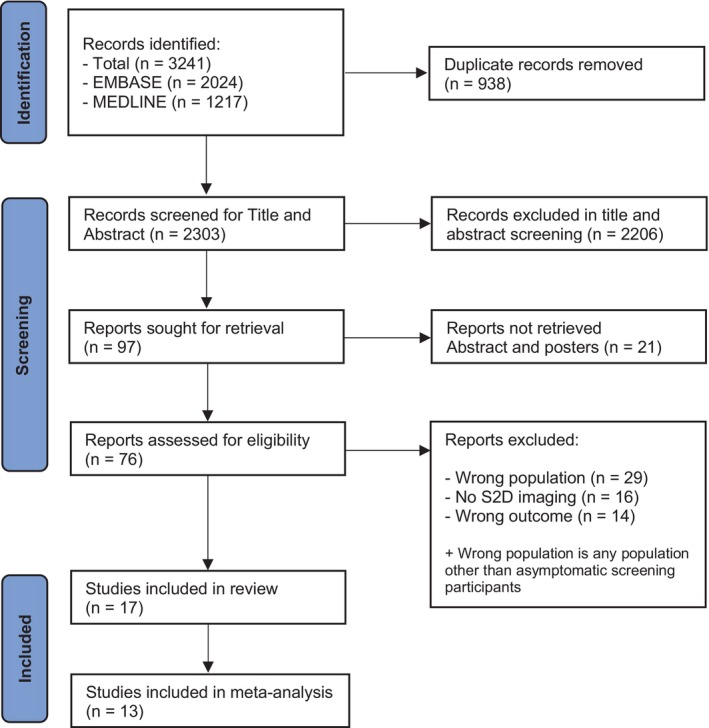
Study selection. Identification of records and the subsequent screening and inclusion process.

A total of 3241 articles were identified using the search strategy described above. After removing duplicates, 2303 articles were eligible for the title and abstract screening. A total of 97 records qualified for full‐text analysis, of which 76 full texts were retrieved, as 21 records were conference posters and abstracts. After full‐text analysis, 17 eligible records^7^, ^17^, ^18^, ^19^, ^20^, ^21^, ^22^, ^23^, ^24^, ^25^, ^26^, ^27^, ^28^, ^29^, ^30^, ^31^, ^32^ were included for the review of which 13 were included in the meta‐analysis.^7^, ^17^, ^18^, ^19^, ^23^, ^24^, ^25^, ^26^, ^27^, ^28^, ^30^, ^31^, ^32^ Four studies^20^, ^21^, ^22^, ^29^ did not provide adequate diagnostic measures or enough data to calculate the CIs or SE and therefore were not included in the meta‐analysis. The details of the identification of articles and the subsequent selection process are described in Figure 1.

Eight studies were conducted in the USA,^17^, ^18^, ^23^, ^24^, ^27^, ^28^, ^29^, ^32^ three studies in Norway,^26^, ^30^, ^31^ and two studies in each of Italy,^7^, ^19^ Germany,^22^, ^25^ and South Korea.^20^, ^21^ All studies recruited asymptomatic participants. Most of the studies' primary target condition was breast cancer^7^, ^17^, ^18^, ^19^, ^22^, ^24^, ^25^, ^26^, ^27^, ^29^, ^30^, ^31^, ^32^; three studies^20^, ^23^, ^28^ described breast micro‐calcification as the primary target condition and one study focused primarily on T1 breast cancer.^21^ All of the studies' reference standards for cancer diagnosis were histopathology and eight studies^19^, ^20^, ^22^, ^23^, ^28^, ^29^, ^30^, ^31^ employed follow‐up as the reference standard for cancer‐free participants. Most studies had a cohort design, eight retrospective^17^, ^18^, ^21^, ^22^, ^23^, ^24^, ^28^, ^29^ and six prospective.^7^, ^19^, ^26^, ^27^, ^30^, ^32^ Two studies^25^, ^31^ were Randomized Control Trials, and one^20^ was a case–control study.

The total population of the included studies was 452,218. The majority of studies (*n* = 15) provided diagnostic performance measures on DBT plus S2D.^7^, ^17^, ^18^, ^19^, ^20^, ^22^, ^24^, ^25^, ^26^, ^27^, ^28^, ^29^, ^30^, ^31^, ^32^ A large number of studies (*n* = 14) reported on the performance of FFDM alone.^7^, ^18^, ^19^, ^20^, ^21^, ^22^, ^23^, ^24^, ^25^, ^26^, ^28^, ^30^, ^31^ Some studies (*n* = 9) reported on the performance of DBT plus FFDM,^17^, ^18^, ^20^, ^22^, ^24^, ^27^, ^29^, ^30^, ^32^ and only three studies provided measures on S2D alone.^20^, ^21^, ^23^ Thirteen studies were included in the meta‐analysis. Meta‐analysis was conducted for DBT plus S2D, FFDM alone, and DBT plus FFDM. S2D alone diagnostic performance measures were not pooled due to inadequate data. Meta‐analysis was performed for CDR and specificity only. Meta‐analysis was not performed for sensitivity due to the low number of studies reporting on it. Study and population characteristics are described in Table 1.

**TABLE 1:** Study and population characteristics.

Study	Country	Study design	Indication for imaging	Cohort size	Imaging combinations	Age (median/mean)	Primary target condition	Reference cancer/cancer free
Bernardi et al.^7^	Italy	Prospective cohort	Screening program	9672	DBT + S2D/FFDM	58/NA	Breast cancer	Biopsy
Zuckerman et al.^32^	USA	Prospective cohort	Screening program	20,937	DBT + S2D/DBT + FFDM	NA/56.7	Breast cancer	Biopsy
Choi et al.^21^ ^,^ ^a^	South Korea	Retrospective cohort	Due to screening findings	214	FFDM/S2D	NA/51.7	Breast cancer T1	Biopsy
Aujero et al.^18^	USA	Retrospective cohort	Screening program	78,810	DBT + S2D/DBT + FFDM/FFDM	NA/56.5	Breast cancer	Biopsy
Freer et al.^24^	USA	Retrospective cohort	Screening program	31,979	DBT + S2D/DBT + FFDM/FFDM	NA	Breast cancer	Biopsy
Ambinder et al.^17^	USA	Retrospective cohort	Screening program	22,535	DBT + S2D/DBT + FFDM	NA	Breast cancer	Biopsy
Hofvind et al.^26^	Norway	Prospective cohort	Screening program	98,927	DBT + S2D/FFDM	59/59.3	Breast cancer	Biopsy
Lai et al.^28^ ^,^ ^b^	USA	Retrospective cohort	Due to screening findings	92	DBT + S2D/FFDM	NA/59	Breast Microcalcificiations	Biopsy/2 years follow up
Choi et al.^20^ ^,^ ^a^ ^,^ ^b^	South Korea	Case–control	Due to screening findings	99	DBT + S2D/DBT + FFDM/FFDM/S2D	NA/50.9	Breast Microcalcificiations	Biopsy/2 years follow up
Clauser et al.^22^ ^,^ ^a^	Germany	Retrospective cohort	Due to screening findings	205	DBT + S2D/DBT + FFDM/FFDM	NA/56.2	Breast cancer	Biopsy/1 year follow up
Hofvind et al.^31^	Norway	Randomized clinical trial	Screening program	28,749	DBT + S2D/FFDM	NA	Breast cancer	Biopsy/2 years follow up
Simon et al.^29^ ^,^ ^a^	USA	Retrospective cohort	Due to screening findings	189	DBT + S2D/DBT + FFDM	NA	Breast cancer	Biopsy/1 year follow up
Skaane et al.^30^	Norway	Prospective trial	Screening program	24,301	DBT + S2D/DBT + FFDM/FFDM	NA/59.1	Breast cancer	Biopsy/2 years follow up
Dodelzon et al.^23^ ^,^ ^b^	USA	Retrospective cohort	Due to screening findings	160	S2D/FFDM	57.0/57	Breast Microcalcificiations	Biopsy/2 years follow up
Caumo et al.^19^	Italy	Prospective cohort	Repeat screening program	34,638	DBT + S2D/FFDM	NA	Breast cancer	Biopsy/2 years follow up
Heindel et al.^25^	Germany	Randomized clinical trial	Screening program	99,689	DBT + S2D/FFDM	NA	Breast cancer	Biopsy
Huang et al.^27^	USA	Prospective cohort	Screening program	1022	DBT + S2D/DBT + FFDM	60/NA	Breast cancer	Biopsy

Abbreviation: NA, not available.

^a^ Excluded from meta‐analysis due to inadequate measures.

^b^ Only data on malignant microcalcifications were extracted.

### 3.1 Comparing DBT plus S2D to FFDM alone

The diagnostic performance measures are described in Table 2. Sensitivity is defined as the estimate as calculated and reported in the individual studies. For all but one of these, sensitivity was calculated as the proportion of cancers identified in a retrospective rereading exercise. For the prospective Oslo trial sensitivity was calculated as the number of cancers screened and detected divided by the total number of screens detected cancers plus those arising symptomatically in the 2 years following the screen.^30^

**TABLE 2:** Diagnostic accuracy measures.

Measures (95 CI %)	DBT ± S2D			FFDM			DBT ± FFDM			S2D		
	CDR/1000	Specificity %	Sensitivity %	CDR/1000	Specificity %	Sensitivity %	CDR/1000	Specificity %	Sensitivity %	CDR/1000	Specificity %	Sensitivity %
Study												
Bernardi et al.^7^	8.8 (7–10.8)	95.5 (95.1–95.9)	_	6.3 (4.8–8.1)	96.6 (96.2–96.9)	_	_	_	_	_	_	_
Zuckerman et al.^32^	5.0 (3.1–6.9)	93.3 (92.7–94.0)	_	_	_	_	5.5 (4.3–6.6)	91.7 (91.3–92.2)	_	_	_	_
Choi et al.^21^ ^,^ ^a^	_	_	_	_	85 (na)	67 (na)	_	_	_	_	91 (na)	68 (na)
Aujero et al.^18^	6.1 (4.9–7.3)	96.3 (96.0–96.6)	_	5.3 (4.5–6.1)	91.8 (91.5–92.1)	_	6.3 (5.5–7.2)	94.8 (94.5–95.1)	_	_	_	_
Freer et al.^24^	5.9 (4.3–7.4)	94.8 (94.3–95.2)	_	5.9 (4.9–6.9)	91.9 (91.5–92.2)	_	6.9 (1.8–11.9)	93.7 (92.2–95.2)	_	_	_	_
Ambinder et al.^17^	5.6 (4.4–6.8)	93.5 (93.1–93.9)	_	_	_	_	5.2 (3.6–6.8)	92.8 (92.2–93.4)	_	_	_	_
Hofvind et al.^26^	9.4 (8.4–10.3)	97.5 (97.4–97.7)	_	6.1 (5.5–6.8)	97.3 (97.2–97.4)	_	_	_	_	_	_	_
Lai et al.^28^ ^,^ ^b^	_	95.0 (88.0–99.0)	94 (86–98)	_	98.0 (91.0–100)	92 (83–97)	_	_	_	_	_	_
Choi et al.^20^ ^,^ ^a^ ^,^ ^b^	_	100 (na)	91 (na)	_	100 (na)	83 (na)	_	100 (na)	91 (na)	_	100 (na)	87 (na)
Clauser et al.^22^ ^,^ ^a^	_	67.7 (na)	83.1 (na)	_	60.2 (na)	72.5 (na)	_	64.6 (na)	82.6 (na)	_	_	_
Hofvind et al.^31^	6.6 (5.3–7.9)	97.6 (97.3–97.8)	_	6.1 (4.8–7.3)	96.6 (96.3–96.9)	_	_	_	_	_	_	_
Simon et al.^29^ ^,^ ^a^	_	96 (na)	83 (na)	_	_	_	_	94 (na)	84 (na)	_	_	_
Skaane et al.^30^	_	95.4 (95.1–95.7)	69 (63.6–74.4)	_	94.2 (93.9–94.5)	54.1 (48.3–59.9)	_	94.9 (94.6–95.2)	70.5 (65.2–75.8)	_	_	_
Dodelzon et al.^23^ ^,^ ^b^	_	_	_	_	98.0 (92.0–100)	73.0 (61.0–83.0)	_	_	_	_	91 (83–96)	77 (66–86)
Caumo et al.^19^	8.1 (6.7–9.5)	97.4 (97.1–97.6)	_	3.5 (2.6–4.4)	96.7 (96.4–96.9)	_	_	_	_	_	_	_
Heindel et al.^25^	8.4 (7.6–9.2)	95.9 (95.7–96.0)	_	6.1 (5.5–6.8)	95.5 (95.4–95.7)	_	_	_	_	_	_	_
Huang et al.^27^	6.8 (1.8–11.9)	93.3 (91.8–94.8)	_	_	_	_	7.8 (2.4–13.2)	92.8 (91.2–94.4)	_	_	_	_

Abbreviation: na, not available.

^a^ Not included in the meta‐analysis.

^b^ Included measures for malignant microcalcifications.

Seven studies^7^, ^18^, ^19^, ^24^, ^25^, ^26^, ^31^ compared the performance of DBT plus S2D to FFDM alone in terms of CDR and FPR (specificities were calculated from the latter). All these studies show higher or equal CDRs for DBT plus S2D compared to FFDM alone. The ranges for CDRs for DBT plus S2D and FFDM alone in these studies were (9.4/1000–5.9/1000) and (6.3/1000–3.5/1000), respectively. Six studies^18^, ^19^, ^24^, ^25^, ^26^, ^31^ show higher specificity for DBT plus S2D. The specificity ranges are (97.6–94.8) and (97.3–91.8) for DBT plus S2D and FFDM alone, respectively. A recent large RCT with almost 100,000 participants concluded a higher CDR and specificity (lower FPR) for the DBT plus S2D compared to FFDM alone.^25^

Four studies^20^, ^22^, ^28^, ^30^ reported on DBT plus S2D and FFDM alone in terms of specificity and sensitivity. Three studies^22^, ^28^, ^30^ reported higher sensitivity and three studies reported higher or equal specificity for DBT plus S2D.^20^, ^22^, ^30^

### 3.2 Comparing DBT plus S2D and DBT plus FFDM

Five studies^17^, ^18^, ^24^, ^27^, ^32^ compared the performance of DBT plus S2D and DBT plus FFDM in terms of CDRs and FPRs (specificities calculated from the latter as before). Four of the studies^18^, ^24^, ^27^, ^32^ concluded a higher CDR for DBT plus FFDM compared to DBT plus S2D with a range of (7.8/1000–5.2/1000) and (6.8/1000–5.0/1000), respectively. However, all the studies reported a higher specificity for DBT plus S2D. Four studies^20^, ^22^, ^29^, ^30^ compared the performance of the imaging combinations in terms of sensitivity and specificity. All reported higher or equal specificity and only two studies^20^, ^22^ reported higher or equal sensitivity for DBT plus S2D compared to DBT plus FFDM.

### 3.3 Comparing S2D alone and FFDM alone

Three studies^20^, ^21^, ^23^ reported on the sensitivity and specificity of S2D alone and FFDM alone. One study^20^ showed identical specificity and higher sensitivity in favor of S2D. Another study^23^ reported higher specificity for FFDM and higher sensitivity for S2D. One study^21^ reported higher specificity in favor of S2D and similar sensitivity.

### 3.4 Meta‐analysis of within‐study comparisons

**FIGURE 2:**
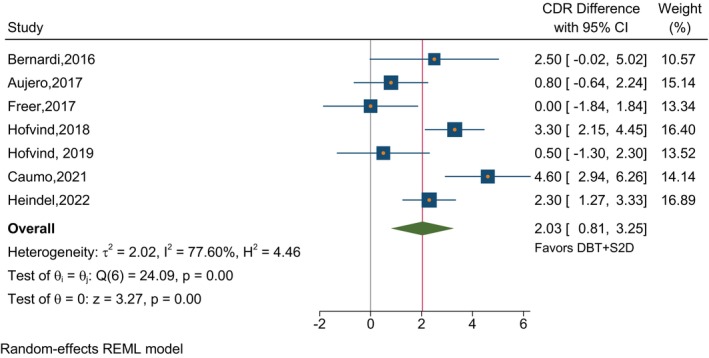
CDR differences in studies reporting on DBT plus S2D and FFDM alone. The absolute estimated CDR difference was 2.03 (95% CI 0.81–3.25) per thousand in favor of DBT + S2D within individual studies comparing DBT + S2D and FFDM.

**FIGURE 3:**
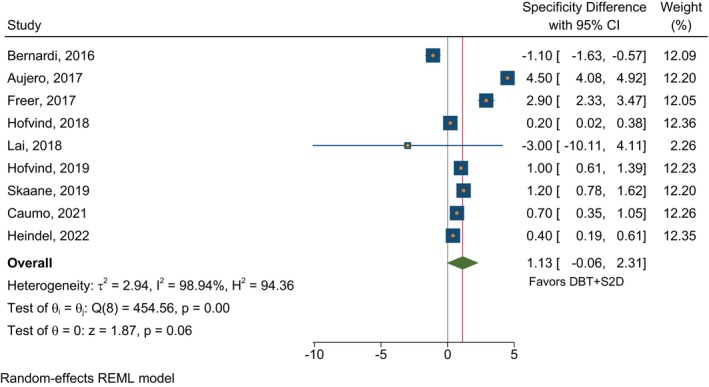
Specificity differences in studies reporting on DBT plus S2D and FFDM alone. The absolute estimated specificity difference was 1.13 (95% CI −0.06 to 2.31) in favor of DBT + S2D within individual studies comparing DBT + S2D and FFDM. However, with a borderline significance (*p* = .06).

**FIGURE 4:**
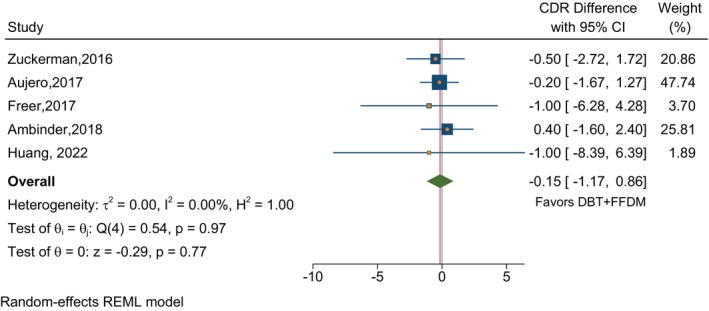
CDR differences within studies reporting on DBT plus S2D and DBT plus FFDM. The absolute estimated CDR difference was −0.15 (95% CI −1.17 to 0.86), a very small and non‐significant difference in CDR between DBT + S2D and DBT + FFDM in favor of DBT + FFDM.

**FIGURE 5:**
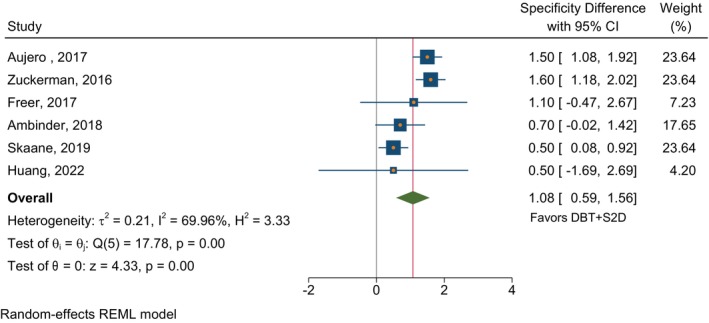
Specificity differences in studies reporting on DBT plus S2D and DBT plus FFDM. The absolute estimated specificity difference was 1.08 (95% CI 0.59–1.56) in favor of DBT + S2D within individual studies comparing DBT + S2D and DBT + FFDM.

In studies that compared DBT plus S2D to FFDM alone in terms of CDR, the absolute estimated CDR difference was 2.03 (95% CI 0.81–3.25) per thousand in favor of DBT plus S2D (Figure 2). The comparison of specificities in Figure 3 showed higher specificity with DBT plus S2D of borderline significance (*p* = 0.06) with a combined difference of 1.13 (95% CI −0.06 to 2.31). For comparison of DBT plus S2D with DBT plus FFDM, there was a very small and non‐significant difference in CDR (Figure 4). There was significantly higher specificity for DBT plus S2D compared to DBT plus FFDM (*p* <.001), with a difference of 1.08 (95% CI 0.59–1.56) in favor of DBT plus S2D (Figure 5). Despite the average differences being similar, the specificity comparison for DBT plus S2D versus FFDM alone was not significant due to the very strong heterogeneity among studies for this comparison‐ three of the studies reporting this comparison had results in the opposite direction.

### 3.5 Meta‐analysis of individual performance measures

These are reported in Supplementary Appendix 2. Specificity results are consistent with those above. Results with respect to CDR are heavily confounded with study population.

## 4 DISCUSSION

We reviewed the published evidence and conducted a meta‐analysis finding no evidence that there was any loss of detection capability or specificity as a result of using S2D when combined with DBT. Combining DBT with FFDM to improve cancer detection in breast cancer screening exposes women to a higher radiation dose.^4^, ^8^ S2D is derived from reconstructed DBT, thus DBT plus S2D exposes women to less radiation than DBT plus FFDM. This systematic review and meta‐analysis aimed to evaluate the performance of S2D plus DBT and compare it to the performance of FFDM alone or plus DBT. The review only identified two studies that evaluated S2D alone, limiting its inclusion in the meta‐analysis. To evaluate the significance of the findings—with no confounding by population factors, the absolute differences in CDR and specificity within individual studies were measured and meta‐analyzed (Figures 2, 3, 4, 5). Our findings indicate that DBT plus S2D is associated with a higher CDR compared to FFDM alone, the estimated absolute difference in CDR per thousand screened being 2.03 (95% CI 0.81–3.25). It should be noted, however, that there was no improvement in CDR for DBT plus S2D compared to DBT plus FFDM. In addition, DBT plus S2D resulted in a higher specificity compared to DBT plus FFDM with an absolute difference of 1.08 (95% CI 0.59–1.56). A similar result was observed for specificity for DBT plus FFDM compared to FFDM alone, however, this fell just short of statistical significance, due to the heterogeneity of results among studies.

The differences observed may be clinically relevant. For DBT plus S2D compared to FFDM alone, we observed an increased detection of approximately 2 per thousand. In the UK program, for example, this would constitute more than a 20% increase in detection rates. While absolute increases such as this cannot be assumed to be generalisable, it is of note that the highest increases were in studies in Italy and Norway, countries with a high incidence of breast cancer typical in high‐income countries. An absolute increase in specificity of the order of 1% might well be important. In the UK program, this would amount to approximately 20,000 fewer false positive recalls per year.^33^

In a previous meta‐analysis by Albousi et al., the estimated CDR for DBT plus S2D was 7.40 (95% CI 6.49–8.37), higher than DBT plus FFDM and FFDM alone, 6.3 (95%CI 5.62–7.14) and 4.68 (95% CI 4.28–5.11), respectively. Comparing DBT plus S2D to FFDM alone revealed a significant difference. However, the estimated measures for the different imaging combinations were the result of pooling findings from different studies.^34^ Our findings were closely consistent with Albousi et al. as DBT plus S2D resulted in a significantly higher CDR than FFDM alone.

Another meta‐analysis by Heywang‐Köbrunner et al. concluded a statistically higher CDR for DBT plus S2D when compared to FFDM alone with a relative ratio (RR) of 1.35 (95% CI 1.20–1.52). This was consistent with a previous meta‐analysis by Giampietro et al. that showed similar results with an RR of 1.38 (95% CI 1.24–1.54).^35^, ^36^ In our review, the differences in diagnostic measures between different imaging combinations were calculated within individual studies and then pooled. In addition, our review compared the performance of DBT plus S2D not only to FFDM alone but also to DBT plus FFDM. Our results showed no significant differences between DBT plus S2D and DBT plus FFDM with respect to CDR. Nowadays, DBT plus FFDM is recommended by many guidelines to enhance performance in breast cancer screening, leading to greater detection capability but also to higher radiation exposure among screened women.^5^, ^6^ Our findings showed a significant difference in the CDR in favor of DBT plus S2D when compared to FFDM alone and a significant difference in specificity in favor of DBT plus S2D when compared to DBT plus FFDM.

This review has several limitations; measures on S2D alone were not included in the meta‐analysis due to insufficient data. Similarly, the sensitivity of the different imaging combinations was not meta‐analyzed for the same reason. Where available they were reported in tables. Most of the included studies were not randomized which might increase the risk of bias.

Significant heterogeneity among study results was observed for CDR and specificity in comparison of DBT plus S2D with FFDM alone, and for specificity in comparison of DBT plus S2D with DBT plus FFDM. For the latter, although there was significant variation among results, they were all in the same direction, an improvement in specificity with use of DBT plus S2D. For the comparison of CDR between DBT plus S2D and FFDM alone, it is worth noting that the most negative result was from a retrospective cohort^24^ and the most positive result from a prospective cohort study.^19^ For the comparison of specificity between DBT plus S2D and FFDM, of the four studies, with results furthest from the overall estimate, three were retrospective cohort studies^18^, ^24^, ^28^ and one was a prospective cohort study.^7^ The most negative (and most uncertain) result was from a study for which the disease endpoint was not all breast cancer but microcalcifications.^28^ The overall results, however, would be little changed by removing the “outliers.”

In addition, the review relied on the CDR and specificity as measures of diagnostic performance, since these were most generally reported or calculable from the published data. Other measures such as recall rates and positive predictive values were not included within the scope of this review. Our review included studies conducted in different countries with variable recommendations and guidelines, which might affect the interpretation of the results. While the histopathological diagnosis was the reference standard in all studies, only eight studies identified a specific follow‐up period for those screened negative, six of them with a 2‐year follow‐up and two with a 1‐year follow‐up. Moreover, the performance of different imagining combinations was not evaluated in different cancer types and stages.

In conclusion, this review demonstrates that DBT plus S2D performs comparably to DBT plus FFDM and is associated with higher CDR compared to FFDM alone. The implementation of DBT plus S2D in breast cancer screening is deemed safe and maintains diagnostic accuracy while mitigating the risk of higher radiation associated with DBT plus FFDM. Future research should focus on evaluating the performance of DBT plus S2D across various mammographic features and at detecting tumors in different stages and types.

## AUTHOR CONTRIBUTIONS

**Wasim Hamad:** Data curation; formal analysis; investigation; methodology; project administration; resources; visualization; writing – original draft; writing – review and editing. **Michael J. Michell:** Conceptualization; supervision; writing – review and editing. **Jonathan P. Myles:** Conceptualization; data curation; writing – review and editing. **Fiona J. Gilbert:** Supervision; writing – review and editing. **Yan Chen:** Writing – review and editing. **Huajie Jin:** Writing – review and editing. **John Loveland:** Writing – review and editing. **Mark Halling‐Brown:** Writing – review and editing. **Keshthra Satchithananda:** Writing – review and editing. **Juliet Morel:** Writing – review and editing. **Asif Iqbal:** Conceptualization; supervision; writing – review and editing. **Rema Wasan:** Writing – review and editing. **Caroline Taylor:** Writing – review and editing. **Nisha Sharma:** Writing – review and editing. **Alexandra Valencia:** Writing – review and editing. **Will Teh:** Writing – review and editing. **Faisal Majid:** Writing – review and editing. **Ronald M De Visser** **:** Writing – review and editing. **Stephen W. Duffy:** Conceptualization; methodology; resources; supervision; validation; writing – review and editing. [Correction added on 29 November 2024, after first online publication: “Conceptualization and supervision” have been added to the contributions of Asif Iqbal.].

## CONFLICT OF INTEREST STATEMENT

The authors declare no conflict of interest exists.

## Supporting information


**Data S1.** Supporting Information.

## Data Availability

The data that support the findings of this study are available from the corresponding author upon request.
