# Supplementary material for: Diagnostic performance of tomosynthesis plus synthetic mammography versus full‐field digital mammography with or without tomosynthesis in breast cancer screening: A systematic review and meta‐analysis
Source: Int J Cancer. 2024 Oct 12;156(5):969–79. doi: 10.1002/ijc.35217 (PMC11701408; doi:10.1002/ijc.35217)
Supplement: Supplementary file 1 — Data S1. Supporting Information. [file IJC-156-969-s001.pdf]

Diagnostic performance of Tomosynthesis plus Synthetic Mammography vs Full-field Digital Mammography with or without Tomosynthesis in breast cancer screening: A systematic review and meta-analysis

Wasim Hamad, Michael J. Michell, Jonathan P. Myles, Fiona J. Gilbert, Yan Chen, Huajie Jin, John Loveland, Mark Halling-Brown, Keshthra Satchithananda, Juliet Morel, Asif Iqbal, Rema Wasan, Caroline Taylor, Nisha Sharma, Alexandra Valencia, Will The, Faisal Majid, Ronnie Devisser, Stephen W. Duffy.

Table of Contents

Supplementary Appendix 1: Search Strategy *2*

Table S1: Search Strategy 2

Supplementary Appendix 2: Meta-analysis for the different imaging combinations *3*

Table S2: Meta-Analysis Summary of Cancer Detection Rate (CDR) and Specificity for DBT+S2D, FFDM Alone, and DBT+FFDM 3

Figure S1: Forest plot for the CDRs of DBT plus S2D 4
Figure S2: Forest plot for the CDRs of FFDM alone 4
Figure S3: Forest plot for the CDRs of DBT plus FFDM 5
Figure S4: Forest plot for the specificity of DBT plus S2D 5
Figure S5: Forest plot for the specificity of FFDM alone 6
Figure S6: Forest plot for the specificity of DBT plus FFDM 6

Supplementary Appendix 3: Calculation of standard errors and 95% confidence intervals from published numbers *7*

References *7*

Supplementary Appendix 1: Search Strategy

Table S1: Search Strategy

Number	Term
#1	‘digital mammography’/exp
#2	(‘digital mammography’ OR ‘digital mammogram’ OR ‘digital mammogram*’ OR ‘full field digital mammography’ OR ‘full field digital mammogram’ OR ‘full field digital mamogra*’ OR ‘ffdm’)
#3	#1 OR #2
#4	(‘synthetic mammography’ OR ‘synthetic mammogram’ OR ‘synthetic mammogram*’ OR ‘sm’)
#5	(‘digital breast tomosynthesis’/exp OR ‘breast tomosynthesis system’/exp)
#6	(‘digital breast tomosynthesis’ OR ‘breast tomosynthesis system’ OR ‘dbt’ OR ‘digital breast tomosynthesis unit’ OR ‘digital breast tomosynthesis system’ OR ‘breast tomosynthesis unit’ OR ‘breast tomosynth*’ OR ‘digital breast tomosynth*’)
#7	#5 OR #6
#8	#4 OR #7
#9	#3 AND #8

Supplementary Appendix 2: Meta-analysis for the different imaging combinations

Meta-analysis was conducted for DBT plus S2D, FFDM alone, and DBT plus FFDM. The estimated pooled results were conducted for CDRs and Specificities. Meta-analysis was not performed for S2D alone nor for sensitivities. Estimated results are summarised in Table S2.

The estimated CDRs per a thousand for DBT plus S2D, FFDM alone and DBT plus FFDM were 7.14 (95%CI: 6.14-8.14), 5.58 (95%CI: 4.83-6.33) and 5.91 (95%CI: 5.27-6.56), respectively. The estimated specificities for DBT plus S2D, FFDM alone, and DBT plus FFDM were 95.54% (95% CI 94.61% - 96.47%), 95.40% (95% CI 93.98% – 96.83%) and 93.48% (95 % CI 92.39% – 94.57%). Forest plots for each modality and diagnostic performance measure are below (Figure S1- S6).

Table S2: Meta-Analysis Summary of Cancer Detection Rate (CDR) and Specificity for DBT+S2D, FFDM Alone, and DBT+FFDM

Measures (n)	DBT+S2D (95% CI)	FFDM alone (95% CI)	DBT+FFDM (95% CI)
**CDR /1000**	7.14 (6.14-8.14) (10)	5.58 (4.83-6.33) (7)	5.91 (5.27-6.56) (5)
**Specificity %**	95.54 (94.61-96.47) (12)	95.40 (93.98-96.83) (10)	93.48 (92.39-94.57) (6)

(n) Number of studies pooled to generate the measure

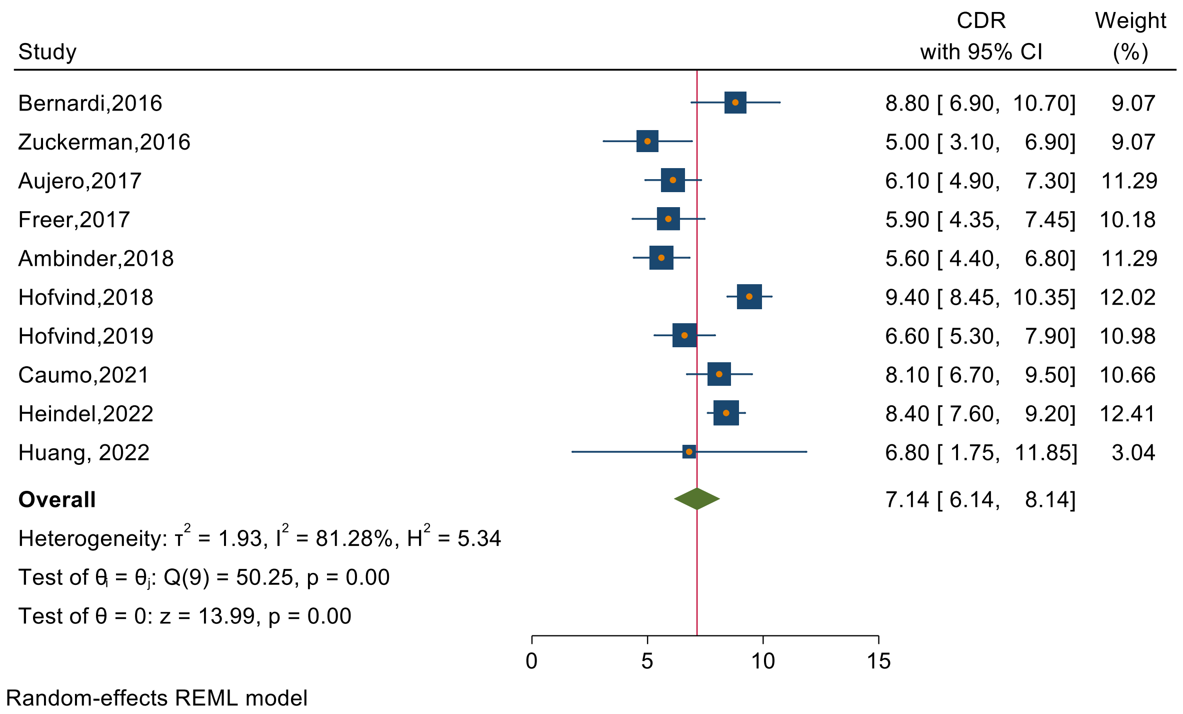

Figure S1: Forest plot for the CDRs of DBT plus S2D

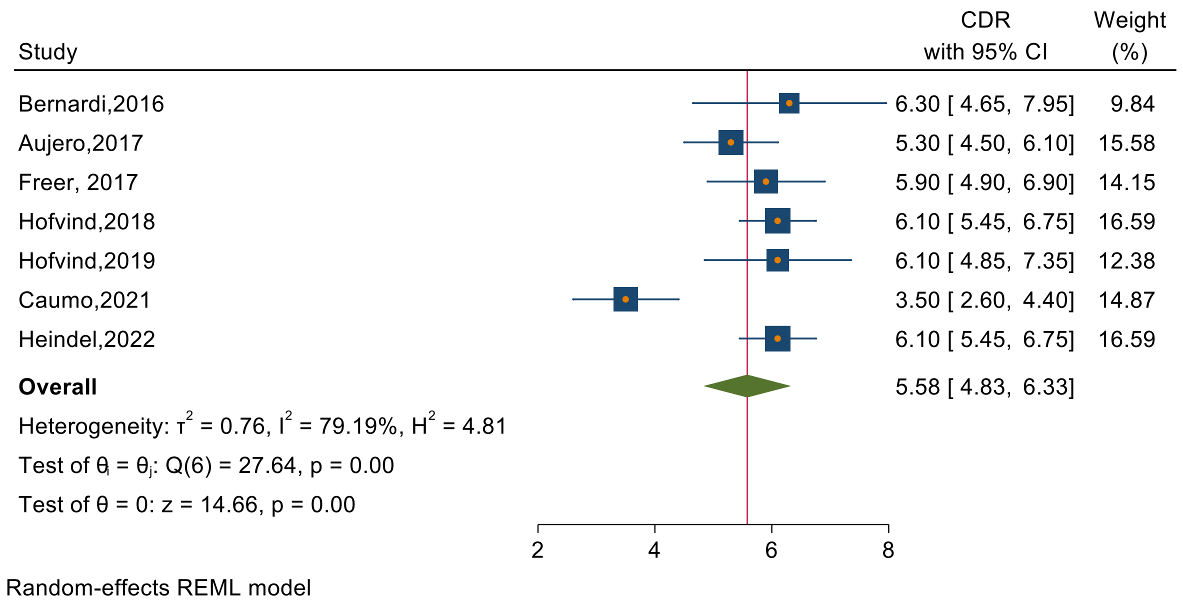

Figure S2: Forest plot for the CDRs of FFDM alone

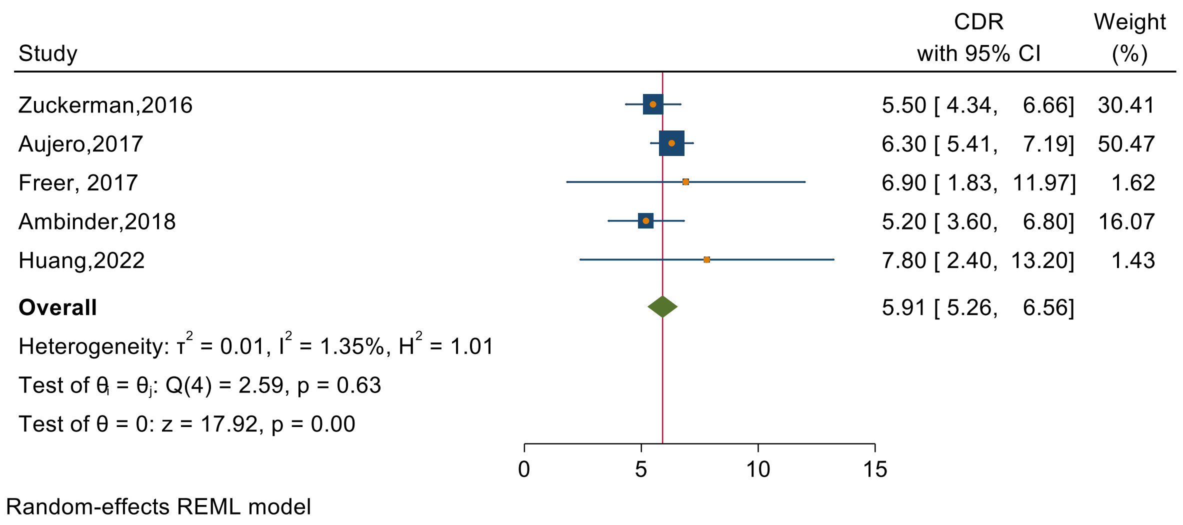

Figure S3: Forest plot for the CDRs of DBT plus FFDM

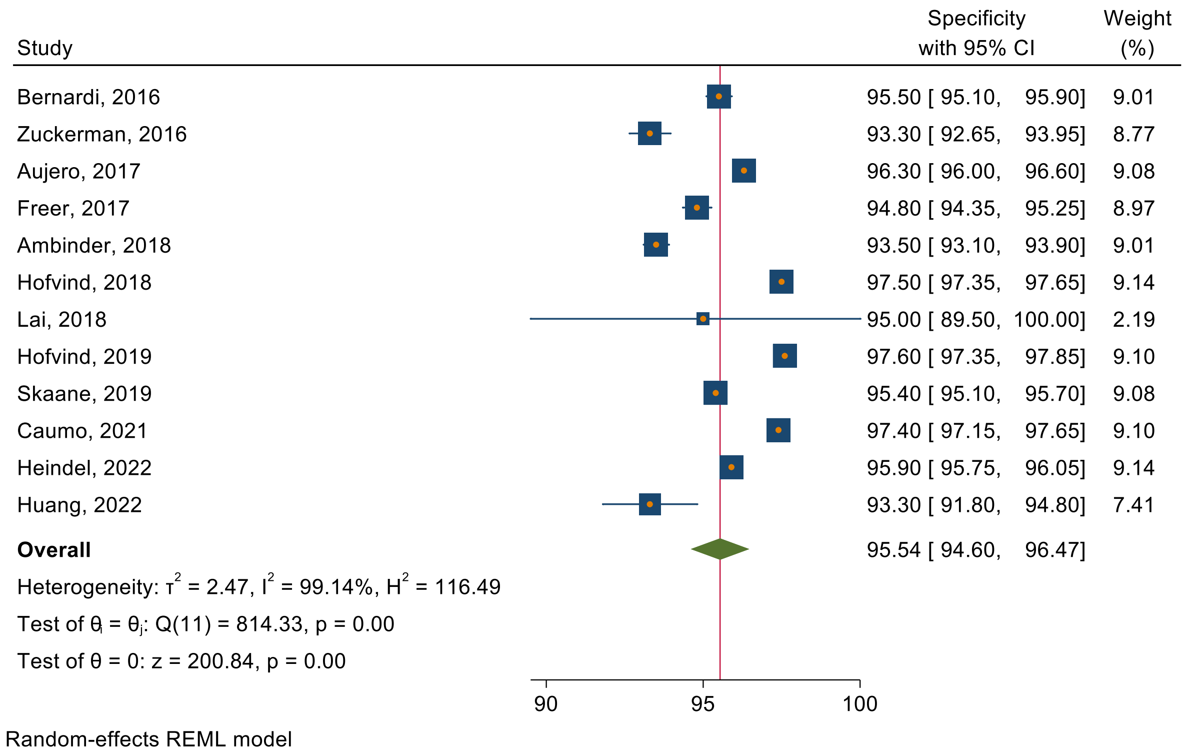

Figure S4: Forest plot for the specificity of DBT plus S2D

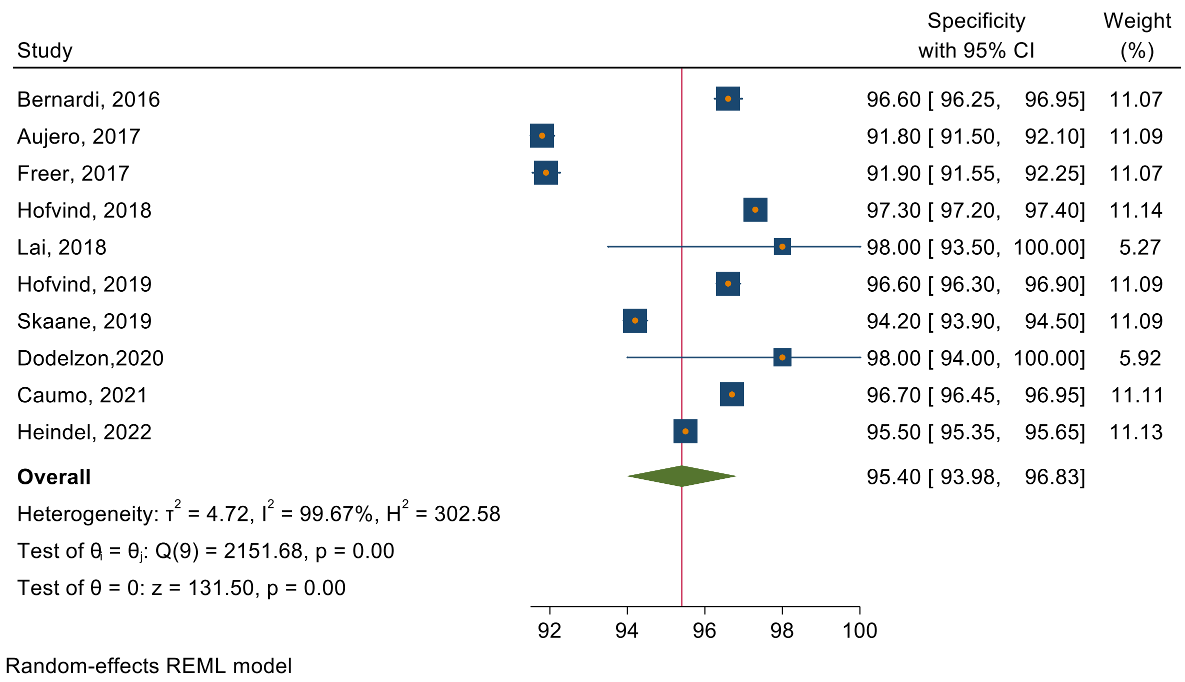

Figure S5: Forest plot for the specificity of FFDM alone

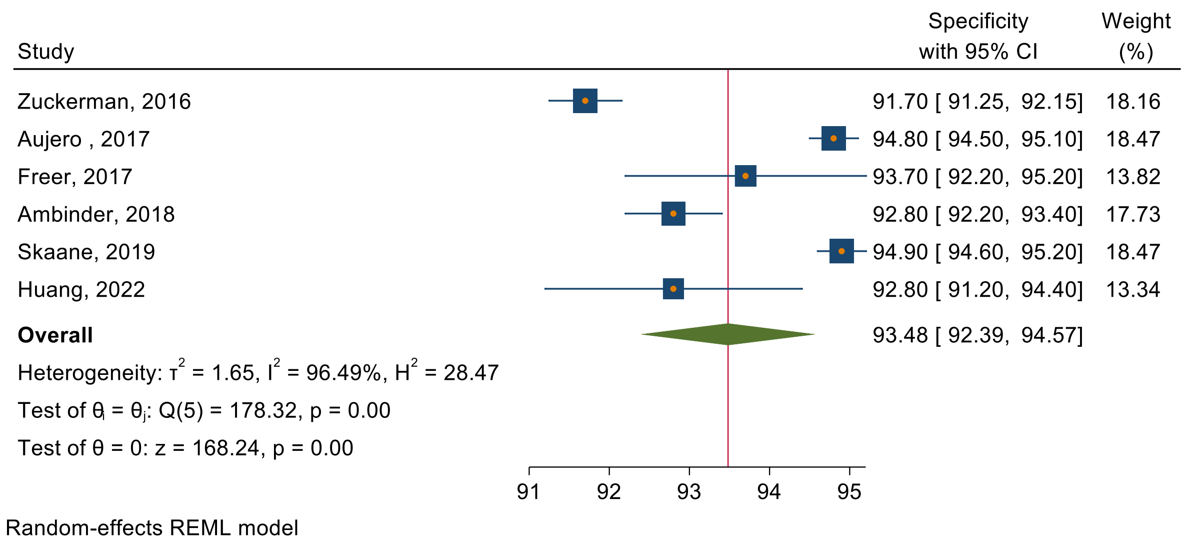

Figure S6: Forest plot for the specificity of DBT plus FFDM

Supplementary Appendix 3: Calculation of standard errors and 95% confidence intervals from published numbers

Consider the example of standard errors on specificity in Skaane et al (31). Skaane et al report that the specificity for FFDM was 94.2% (1,388 false positives in 24020 screenees without breast cancer). On this basis, assuming the number of false positives to be binomially distributed, the standard error is estimated as

$$SE = \sqrt{\frac{0.942 \times 0.058}{24020}} = 0.0015$$

or 0.15%. We then calculated the 95% confidence interval as

$$94.2 \pm 1.96 \times 0.15$$

This gives (93.9-94.5) as the 95% confidence interval.

References

31. Skaane P, Bandos AI, Niklason LT, Sebuodegard S, Osteras BH, Gullien R, et al. Digital Mammography versus Digital Mammography Plus Tomosynthesis in Breast Cancer Screening: The Oslo Tomosynthesis Screening Trial. Radiology. 2019;291(1):23-30
